# Bella and Charlie Are Not the Problem—It’s Us: The Real Causes of Wildlife Rescue in NSW

**DOI:** 10.3390/ani16142174

**Published:** 2026-07-13

**Authors:** Kate Dutton-Regester, Jacquie Rand, Antong Liang

**Affiliations:** 1 Australian Pet Welfare Foundation, Kenmore, QLD 4069, Australia; k.duttonregester@uq.edu.au; 2 School of Veterinary Science, The University of Queensland, Gatton, QLD 4343, Australia; 3 School of Agriculture and Food Sustainability, The University of Queensland, Gatton, QLD 4343, Australia; antongliangec@gmail.com

**Keywords:** rehabilitation records, wildlife, wildlife rescue, threatened species, wildlife mortality, wildlife conservation, cats, dogs

## Abstract

Public discussion in Australia often presents pet cats as a major cause of wildlife decline, and cat management is frequently promoted as a major conservation response. However, this focus may draw attention away from other common and preventable recorded causes of threatened species rescue. Using the NSW Wildlife Rehabilitation Data Dashboard, we present the first statewide analysis of NSW wildlife rehabilitation records to specifically examine threatened species, rather than pooling threatened and non-threatened wildlife together. This dataset represented 52,475 individuals and 158 threatened species rescued from 2013 to 2024 in NSW. Rehabilitation data represent only animals that are found, reported, and brought into care, mostly in human-frequented environments, and should not be interpreted as estimates of total wildlife mortality, predation pressure, or ecological impact beyond the rehabilitation dataset. Outcomes were often poor: 24.1% were released, 58.5% died, and 17.5% had other recorded outcomes. The largest recorded category was Unknown (21.9%), which should not be interpreted as absence of cause, but as cases where a specific cause could not be assigned at the time of reporting to the dashboard. Among identified recorded causes, the most common were Entanglement (11.6%), Weather Event (11.4%), Abandoned/Orphaned (10.1%), Unsuitable Environment (7.3%), and Motor-Vehicle Collision (5.8%). Together, Unknown and these five identified causes accounted for 68.1% of recorded threatened species rescues. By comparison, all animal attacks combined accounted for 4.4%; dog-related rescues were more than three times as frequent as cat-related rescues, with cat-related rescues comprising 0.6%. These findings show that cat attacks formed a small proportion of recorded rescues, although overall ecological impact cannot be determined from these data. Prevention efforts targeting common and preventable recorded causes may help reduce the number of threatened animals entering care.

## Simple Summary

Public debate in Australia often focuses on pet cats as a major threat to wildlife. While cats can affect wildlife in some situations, this study looked at what brings threatened animals into wildlife care in New South Wales. We analyzed 11 years of records from the NSW Wildlife Rehabilitation Data Dashboard, covering 52,475 rescued animals from 158 threatened species. This is the first statewide study to look specifically at threatened species in these rescue records. Importantly, these records only include animals that were found, reported, and brought into care, mostly in places where people live, work, travel, or visit. They do not show all wildlife deaths or the total impact of any threat beyond the rehabilitation dataset. Outcomes for rescued threatened animals were often poor: 24.1% were released alive, 58.5% died, and 17.5% had another recorded outcome. The most common recorded reason for rescue was Unknown, followed by Entanglement, Weather Event, Abandoned or Orphaned young, Unsuitable Environment, and Motor-Vehicle Collisions. Together, these accounted for 68% of threatened species rescues. Animal attacks made up 4.4% of recorded rescues. Dog attacks were more than three times as common as cat attacks, while cat attacks accounted for 0.6% of threatened species rescues. These findings show that preventing common recorded rescue causes, such as entanglement, heat stress, unsafe environments, and road injuries, could help reduce the number of threatened animals needing care.

## 1. Introduction

In Australia, media reports often present pet cats as a major cause of urban wildlife decline. Cats are frequently described as major ‘killers’, with claims that they are ‘sending our suburbs silent’ and that people should ‘lock up your pet—it’s a killing machine’ [1,2,3,4,5]. Many of these claims are based on modeling and estimates drawn largely from non-peer-reviewed sources and from data collected 32 to 37 years ago, including cat owners’ reports of prey brought home [6,7,8,9,10,11]. This is important because urban cat ownership and containment practices have changed substantially over time; older estimates used in influential modeling were based on approximately 29% of cats being contained [6], whereas recent Australian data indicate that approximately 65% of pet cats are fully contained and a further 24% are contained at night [12]. We do not dispute that cats can affect wildlife in natural environments. However, our concern is that some public and policy claims about urban pet cats rely on extrapolating estimates from studies, models, or ecological settings that may not reflect current urban and periurban conditions, cat ownership practices, containment rates, or prey caught in highly modified environments. For example, widely cited publications such as *‘We need to worry about Bella and Charlie: The impacts of pet cats on Australian wildlife’* [6] use statistical models built from limited and geographically restricted data. These papers promote cat bans and mandatory containment as key solutions. However, evidence is lacking that these measures improve biodiversity outcomes. These policies may also create social and practical barriers, particularly in low-income communities where the costs of cat sterilization, containment, microchipping, and registration can be difficult to meet [13].

Strong public focus on cats risks diverts attention away from other common and preventable impacts on wildlife in urban areas. These include habitat loss and fragmentation, vehicle collisions, extreme weather events, and hazards linked to roads, buildings, and other human structures [14,15]. More up-to-date and comprehensive data are needed to place cat attacks in context and identify where prevention will have most benefit, particularly for threatened species.

Wildlife rehabilitation records provide an important source of information about animals that are found, reported, and brought into care and have been used to identify patterns in morbidity, mortality, human–wildlife interactions, and conservation-relevant threats [16,17,18]. These data can show why animals were rescued, which species were affected, and how outcomes changed over time in highly modified environments. However, rehabilitation records do not include all wildlife injuries, illness, or deaths and are influenced by detection, reporting, and admission processes [17,18]. Whether an animal enters the rehabilitation system depends on several factors, including whether it survives long enough to be found, whether it is visible or accessible to people, whether the event occurs in a place where people live or often visit, and whether the person who finds the animal is able and willing to seek help. These factors are likely to differ across all causes of rescue. For example, some events may be more visible but have lower immediate survival, while others may be less visible or less likely to be reported. The dashboard data cannot show whether each cause is over- or under-represented, or by how much. Therefore, rehabilitation data should not be used to estimate total wildlife deaths in highly modified environments, the true number of animals affected by each cause, or the overall ecological importance of different threats. Instead, these data are valuable for understanding the burden of recorded rescues in places where people and wildlife overlap, and for identifying practical ways to reduce the types of harm that commonly lead animals into care. These findings should not be extrapolated to wildlife injury or mortality in natural environments or remote and less human-frequented environments, where detection, reporting, species composition, and threat processes may differ substantially.

In New South Wales (NSW), more than 100,000 injured, sick, or displaced wild animals are rescued each year by licensed volunteer rehabilitators working under the NSW National Parks and Wildlife Service [19]. These records document the assigned reasons for rescue among animals that enter the rehabilitation system, including extreme weather event, habitat change, vehicle collisions, and domestic-animal attacks [20]. Rehabilitation data therefore allow comparison of recorded causes of rescue, including domestic-animal attacks, vehicle collisions, entanglement, and environmental displacement, while recognizing that these records reflect rescue burden rather than total mortality or ecological impact.

Previous studies of wildlife hospitals across Australia show consistent patterns. Motor-vehicle collisions, orphaned or dependent young, disease, and environmental displacement are usually the leading reasons for admission. In south-east Queensland, admissions to the Australia Zoo Wildlife Hospital were dominated by vehicle collisions (35%), orphaned or dependent young (25%), and disease (8%), with an overall mortality of 57% [21]. Dog attacks accounted for 9% of cases and cat attacks for 5%. A larger dataset from the RSPCA Wildlife Hospital in south-east Queensland showed a similar profile, with orphaned young (31%), motor-vehicle accidents (18%), and disease (9%) most common, and mortality exceeding 60% [22]. Dog attacks accounted for 8% of admissions and cat attacks for 6%. In Victoria, admissions to the Phillip Island Wildlife Clinic were dominated by motor-vehicle trauma (26%) and unknown cause (25%), with a mortality of 70% [23]. In that study, cat and dog attacks did not rank among the top six causes of hospital admission. A Victorian analysis of Wildlife Victoria emergency-response records for threatened species also found that rescue reports were concentrated in human-modified environments and that the leading 10 known causes were entanglement, collision, abnormal location, nuisance, hit by vehicle, found within building, trapped, attack by other animal and dog attack, with cat attacks being the least frequent of the 10 known causes of rescues for threatened species [24].

Within NSW, the first statewide analysis of wildlife rehabilitation data examined 469,553 rescues reported by more than 50 licensed wildlife rescuers between 2013 and 2019, using the NPWS reporting framework now integrated into the NSW Wildlife Rehabilitation Data Dashboard [20,25]. The most common reasons for rescue were vehicle collision (24.3%), abandoned or orphaned young (20.1%), and unsuitable environment (16.8%), with overall mortality of 55%. Threatened species represented 4.6% of rescues but more than 20% of the species recorded, highlighting the conservation value of rehabilitation data. Comparable patterns are evident in NSW annual wildlife rehabilitation summaries, where ‘unsuitable environment’, ‘dependent young’, and ‘collision—motor vehicle’ remain the most frequently reported causes [26,27,28,29,30,31]. Collectively, these studies show a consistent pattern across Australian rehabilitation datasets: many recorded rescues are associated with vehicle collisions, orphaning, environmental displacement, disease, and other hazards linked to human-modified landscapes. Cat attacks are recorded, but they make up a small proportion of reported rescues in these rehabilitation datasets compared to several other recorded causes.

Most previous studies investigating wildlife rescue data have combined all species. As a result, the main causes of rescue for wildlife listed under the Biodiversity Conservation Act 2016 (NSW) remain poorly understood. Understanding which threats most often lead threatened animals into care is important for conservation planning and policy. Rescue records show which recorded causes are most common among animals entering care and how patterns in reported rescues vary across locations and years [22,25]. Because these data combine thousands of volunteer observations over more than a decade, they provide one of the few consistent statewide indicators of human–wildlife interactions through time.

In this study, we analyze threatened species rescues in New South Wales over eleven years (2013–2024), using data from the NSW Wildlife Rehabilitation Data Dashboard [20]. We take a prevention-focused approach. Specifically, we aim to (i) identify the main reasons for rescue among threatened species; (ii) summarize the number of species and individuals affected by each cause; (iii) describe release outcomes for different causes; (iv) assess changes over time; and (v) identify practical prevention actions that may reduce the number of threatened animals entering care and improve outcomes for rescued wildlife and for wildlife rescuers.

## 2. Methodology

### 2.1. Study Area, Data Collection and Preparation

Data for this study were sourced from the NSW Wildlife Rehabilitation Data Dashboard, a publicly accessible resource maintained by the NSW Government [20]. The dashboard collates data submitted annually by all licensed wildlife rescuers operating under the NSW National Parks and Wildlife Service (NPWS) in accordance with the Biodiversity Conservation Act 2016, relevant Codes of Practice, and the NSW Veterinary Practice Act 2003.

The dataset covers the entire state of New South Wales (approximately 800,000 km^2^) and includes records of wildlife rescues conducted under NPWS license conditions between 1 July 2013 and 30 June 2024. Dashboard outputs include the recorded reason for rescue, conservation status, outcome, suburb-level location and reporting organization.

Across the study period, 970,124 wildlife rescues were recorded statewide. From these, all records involving species listed as Critically Endangered, Endangered, or Vulnerable under the Biodiversity Conservation Act 2016 were extracted (*n* = 52,612). Records classified as ‘Exotic/Captive’ were excluded (*n* = 137). After exclusions, the final dataset comprised 52,475 threatened individuals across 1093 species-level records spanning mammals, birds, reptiles, and amphibians.

As these data are publicly available records, no ethical approval was required. Because the dashboard does not provide downloadable files, dashboard-generated summary outputs were manually transcribed into Microsoft Excel (version 16.98) for cleaning and organization. The dashboard automatically generates summary counts according to selected filters, including species, conservation status, reason for rescue, outcome, year, and reporting category. Data were initially entered by A.L. and then checked by K.D.-R. against the dashboard-generated totals. Quality-control checks included comparison of entered values with dashboard totals for all threatened species records, animal groups, reasons for rescue, outcomes, years, and grouped categories. Column totals, subtotals, and category totals were cross-checked against the dashboard-generated summaries, and any discrepancies were corrected before analysis. Because the dashboard provides aggregated summary counts rather than downloadable individual case-level records, we could not independently review each individual rescue record for duplication or reclassification. Similarly, because the study relied on standardized summary outputs generated by the publicly available dashboard rather than manual coding of individual records, inter-observer validation was not applicable. Instead, all manually transcribed summary counts were independently checked against the dashboard-generated totals as described above. Quality control focused on verifying that all transcribed summary counts matched the totals generated by the dashboard for each filter and category. All summary totals used in the analyses were checked against the dashboard-generated counts. Rescue reasons were retained as recorded in the NSW Wildlife Rehabilitation Data Dashboard, except that the four entanglement subcategories—entanglement–other, entanglement–wire, entanglement–netting, and entanglement–marine debris—were combined into a single ‘Entanglement’ category for analyses. These categories were pooled because they represent the same broad mechanism of injury and prevention pathway (Table 1). Throughout the manuscript, categories are interpreted as recorded reasons for rescue in highly modified environments rather than definitive ecological impacts, because the dashboard reflects the category assigned at or soon after rescue and does not include detailed case-level diagnostic information.

Rescue outcomes were grouped into three categories as per the dashboard: released, died, and other. The ‘other’ category included all remaining recorded outcomes, including animals still in care, transferred to another organization, relocated without treatment, reunited with parents, placed into permanent care, or with unknown final status. Seasonal patterns were assessed using standard southern-hemisphere definitions: summer (December–February), autumn (March–May), winter (June–August), and spring (September–November).

### 2.2. Statistical Analysis

All analyses were conducted in R version 4.4.1 [32] using RStudio version 2025.05.1+513 [33]. Descriptive statistics were used to summarize the number and proportion of rescues by species, animal group (mammals, birds, reptiles, amphibians), reason for rescue, and outcome. For each rescue reason category, we calculated the number of threatened species affected, the total number of individuals affected (i.e., the number of rescue records), and the proportion of individuals released. Spearman correlation coefficients were calculated to explore the relationship between the number of species affected and the number of individuals rescued per cause. Rescue reasons were ranked according to frequency (number of rescues), breadth (number of species affected), and death rate (deaths/total rescues per cause), which indicates how severe outcomes were for each rescue reason. Patterns over time and across seasons were assessed using annual summary data. We used linear regression to test whether the number of rescues increased over time for major animal groups and rescue reasons. Linear regression was used to assess the direction and strength of broad temporal trends in annual recorded rescue counts rather than to model count processes or estimate incidence. Given the small number of annual observations (11 years) and the exploratory nature of these analyses, linear regression provided a simple descriptive assessment of change over time. Model assumptions were evaluated through visual inspection of residual plots, which indicated no substantial departures from linearity or homoscedasticity. Results should therefore be interpreted as exploratory trends in recorded rescue numbers rather than formal population-level models.

Because the dashboard provides aggregated rescue counts and suburb/locality-level location information, rather than downloadable case-level records with precise coordinates, standardized survey sites, reporting effort, population denominators, detection histories, or repeated site-level observations, analyses were intentionally descriptive. Trend analyses should therefore be interpreted as exploratory patterns in recorded rescue counts, not estimates of incidence, abundance, occupancy, detection probability, or population-level mortality risk.

Because the dashboard does not provide measures of reporting effort, public awareness, volunteer capacity, search effort, population growth, urbanization intensity, or changes in access to rescue services over time, annual counts could not be standardized for sampling or reporting effort. Temporal analyses should therefore be interpreted as exploratory changes in recorded rescue numbers, not direct evidence of ecological change.

## 3. Results

Across NSW, 967,492 wildlife rescues were recorded during the study period (excluding exotic or captive species, *n* = 2632). For threatened species, after the removal of 137 exotic or captive records, the final dataset included 52,475 threatened animals rescued between 2013 and 2024, representing 158 threatened species. Of these individuals, 12,627 (24%) were successfully released, 30,683 (59%) died in care, and 9165 (18%) had other outcomes. Across the study period, 42 distinct reasons for rescue were recorded.

### 3.1. Threatened Species Rescued

Threatened species rescues were dominated by mammals (87%), followed by birds (9%), reptiles (4%), and amphibians (<0.1%) (Table 2). Most mammal rescues involved the Gray-headed Flying-fox (*Pteropus poliocephalus*), followed by the Koala (*Phascolarctos cinereus*) and Squirrel Glider (*Petaurus norfolcensis*). Bird rescues most often involved the Bush Stone-curlew (*Burhinus grallarius*), Powerful Owl (*Ninox strenua*), and Sooty Tern (*Onychoprion fuscatus*). Reptile rescues were mainly marine turtles, particularly the Green Turtle (Chelonia mydas) and Loggerhead Turtle (*Caretta caretta*). Amphibian rescues were rare (<0.1%) and were mostly Green and Golden Bell Frogs (*Litoria aurea*).

### 3.2. Main Recorded Reasons for Rescue

Across all wildlife rescues (not limited to threatened species), 49% of rescues were recorded as having no known reason for rescue. After Unknown, the most common recorded reasons were motor-vehicle collision, unsuitable environment, abandoned or orphaned young, and fallen from nest or tree. Dog attack ranked ninth overall and cat attack ranked tenth (Table 3).

For threatened species, a small number of causes accounted for most rescues. The ten most common reasons made up 82% of all threatened species rescues (Table 4). The largest category was Unknown cause (22%), affecting 140 threatened species. Only 24% of these animals were released. Entanglement and Weather event (extreme heat) were the second and third-most common causes (12% and 11%, respectively), and both mainly involved Gray-headed Flying-foxes. Release was particularly low for Weather event—extreme heat (1.4%). Other leading causes were abandoned or orphaned young (10%), unsuitable environment (7%), and motor-vehicle collision (6%).

### 3.3. Animal Attacks

All forms of animal attack combined accounted for 4.4% of threatened species rescues (*n* = 2302). Dog attacks made up 2% of rescues (*n* = 1032), followed by suspected or unidentified animal attacks (1%, *n* = 516), bird attacks (0.8%, *n* = 401), cat attacks (0.6%, *n* = 311), same-species attacks (0.1%, *n* = 37), and fox attacks (0.01%, *n* = 5) (Table 5). For comparison, cat attack accounted for 1.8% of all recorded wildlife rescues statewide. Dog attacks were recorded across 36 threatened species, while cat attacks were recorded across 52 threatened species. Release rates were low and similar to the average release rate for all rescues: 26% of dog attack cases (*n* = 271) and 22% of cat attack cases (*n* = 67) resulted in live release. The relative proportion of attack-related rescues differed among mammals, birds, and reptiles, as shown in Table 6.

### 3.4. Leading Causes of Rescue by Animal Group

The main rescue reasons differed across animal groups (Table 6). For mammals, the top three known causes were weather event—extreme heat, entanglement and abandoned/orphaned. Koalas (*Phascolarctos cinereus*, *n* = 11,720) and Gray-headed Flying-foxes (*Pteropus poliocephalus*, *n* = 31,277) represented 94% of mammal rescues. Koala rescues were most commonly linked to disease (chlamydia), followed by unknown cause, motor-vehicle collision, and unsuitable environment. Gray-headed Flying-fox rescues were largely driven by weather event—extreme heat, with other common reasons including unknown cause, entanglement, and motor-vehicle collision. For reptiles, rescues were most often attributed to unknown cause, stranding or haul-out events, and disease or habitat alteration.

### 3.5. Number of Threatened Species Affected and Number of Individuals Affected

The number of species affected differed widely across rescue reasons. Unknown cause affected the greatest number of different threatened species (140), followed by Unsuitable Environment (86), motor-vehicle collisions (83), and Collision—Other (61). Cat attacks ranked fifth in the number of species affected, affecting 52 species, while dog attacks affected 36 species. Across all causes, there was a moderate positive relationship between the number of species affected and the number of individuals rescued (r = 0.69).

### 3.6. Changes over Time

Over the 11-year study period, rescues of threatened birds increased significantly (*p* < 0.01). In contrast, there was no clear long-term change in the number of rescued mammals, reptiles, or amphibians when looked at as broad groups. At the species level, several threatened species showed clear increases in the number of rescues over time. These included seven bird species, one tree-dwelling mammal, five microbat species, and two reptiles (Table 7 and Table 8). Among the 10 most common rescue reasons for threatened species, four increased significantly over time: unsuitable environment, motor-vehicle collision, disease—chlamydia, and electrocution. Disease–chlamydia was almost entirely associated with Koalas, which accounted for 2454 of 2459 (99.8%) of rescues associated with chlamydia. Electrocution records were overwhelmingly associated with Gray-headed Flying-foxes, which accounted for 2106 of 2121 electrocution rescues (99.3%).

Of the entanglement subcategories, entanglement—other, marine debris, and netting all showed significant increases over time. In contrast, rescues recorded as unknown, weather event, abandoned or orphaned young, dog attack, and cat attack did not show a consistent increase or decrease over the study period. Rescues associated with weather events due to extreme heat were highly concentrated in 2019–2020, with 5447 of 5985 records occurring in that year (91.0%). Weather event—fire rescues were also highly concentrated in 2019–2020, with 1320 of 1359 records occurring in that year (97.1%); the remaining rescues in both categories were spread across several years in much smaller numbers. Given this concentration in a single extreme-event year, annual trend estimates were examined for these two weather event categories. For weather event–extreme heat, the estimated annual change was +43.9 rescues per year (95% CI: −325.1 to +412.8; *p* = 0.794). For weather event–fire, the estimated annual change was +11.7 rescues per year (95% CI: −78.1 to +101.5; *p* = 0.776).

## 4. Discussion

This study provides the first statewide summary of NSW wildlife rescue records for threatened species, using 11 years of data (2013–2024) from the NSW Wildlife Rehabilitation Data Dashboard. In total, 52,475 threatened animals were rescued during this period. Only 24% were released back to the wild. Most did not survive: 59% died, and 18% had other outcomes (animals still in care, transferred to another organization, relocated without treatment, reunited with parents, placed into permanent care, or with unknown final status). This 59% death rate suggests that many threatened animals are already in a serious condition by the time they reach care. Most rescues were linked to a small number of main recorded causes. The most common were Unknown (22%), Entanglement (12%), Weather Event (11%), Abandoned or Orphaned (10%), Unsuitable Environment (7%), and Motor-Vehicle Collision (6%). Together, these six categories accounted for 68% of all recorded threatened species rescues. The large Unknown category does not indicate absence of disease, trauma, or other underlying causes, but rather cases where a specific cause could not be assigned within the limits of rescue-based reporting. In contrast, attacks by pet cats and dogs together made up less than 3% of rescues. Dog attacks (2%) were more than three times as common as cat attacks (0.6%). This is notable given the strong public focus on pet cats in wildlife discussions.

Within this threatened species rehabilitation dataset, many of the leading identified recorded categories were associated with pressures linked to human-modified landscapes, including urban design, vegetation clearing, hazardous materials, and roads. These records do not capture all wildlife injury, illness, or death. Instead, they show which causes are most commonly represented among animals entering care. As such, rehabilitation records can help identify prevention opportunities that may reduce the number of threatened animals requiring rescue, including from environmental stress, human-modified landscapes, hazardous materials, roads, and urban infrastructure, while recognizing that they do not measure the total ecological impact of each threat.

Statewide context supports a prevention-first approach. When all NSW wildlife rescues are considered, and not only threatened species, the same pattern appears: nearly half of all rescues are recorded as Unknown, and motor-vehicle collision and unsuitable environment together account for a large additional share. In this broader dataset, dog attack ranked ninth and cat attack tenth, occurring less often than multiple other recorded causes. This broader pattern reinforces the same conclusion within the rehabilitation dataset: the largest reductions in recorded wildlife rescues are most likely to come from addressing the most common and preventable recorded causes, particularly road trauma, displacement into unsafe environments, and hazards associated with roads, buildings, and other human structures and materials. We therefore focus below on the leading recorded causes of threatened species rescues, because they account for more than two-thirds of cases and represent practical opportunities for prevention.

### 4.1. Unknown

‘Unknown’ was the largest recorded category for threatened species rescues and was associated with poor outcomes, with approximately three-quarters of animals either dying or not being released. This category should not be interpreted simply as an absence of cause. Rather, it represents cases where a specific cause could not be assigned at or soon after rescue. Large wildlife hospital studies in Australia also report ‘Unknown’ as one of the most common recorded reasons for hospital admission [21,22,23], with comparable findings from other countries [16,34,35]. This pattern reflects the limits of rescue data rather than the absence of real underlying causes.

In the NSW Wildlife Rehabilitation Data Dashboard, a case is recorded as ‘Unknown’ when the rescuer cannot clearly assign it to another defined category at the time of rescue (Table 1). The recorded category usually remains unchanged unless it is later updated following veterinary assessment. If no clear primary cause can be identified, the case remains classified as ‘Unknown’.

This may occur when animals arrive in poor condition, decline quickly, or die soon after rescue, limiting the opportunity for detailed clinical assessment or diagnostic testing. Previous rehabilitation studies show that condition on arrival is strongly associated with outcome, and that triage and diagnostic decisions in wildlife rehabilitation are often constrained by prognosis, animal welfare considerations, available resources, and feasibility [36,37]. There may be limited opportunity to conduct detailed diagnostic testing before an animal dies or is euthanized on welfare grounds. Even when animals survive longer, diagnostic investigation in wildlife rehabilitation settings may be constrained by prognosis, animal welfare considerations, available facilities, trained personnel, veterinary services, and funding [37]. Importantly, ‘Unknown’ does not mean that nothing was wrong. It means that a specific cause could not be confirmed within the limits of what can realistically be done in a wildlife rescue setting. Many animals in this group likely experienced multiple overlapping conditions that could not be confirmed within the practical constraints of wildlife rescue and rehabilitation. Importantly, an increase in the number of animals recorded as Unknown coincided with heat events.

Disease is under-recorded in this dataset and likely accounts for at least half of the ‘Unknown’ category. In the present dataset, however, disease was largely confined to koalas with the bacterial infection, chlamydia. Chlamydia in koalas is widely recognized, supported by high public awareness, and is often visibly apparent as discharge and inflammation around the eyes or urogenital tract [38]. In other species, disease may be less obvious and more difficult to diagnose. Determining which underlying disease process is causing the animal’s condition, such as degenerative, congenital, metabolic, neoplastic, nutritional, physical or environmental, infection (viral, bacterial, protozoan, fungal, parasitic, etc.), immune, toxic or traumatic, as well as the specific disease within these categories and the specific organ/s affected, often requires extensive and expensive blood tests, imaging, and/or post-mortem examination. Further diagnostic investigation may be constrained in wildlife rescue settings, particularly when animals arrive in critical condition, because triage decisions must prioritize prognosis, animal welfare, available facilities, trained personnel, veterinary support, and resources [36,37]. The relatively low recording of disease outside koalas therefore likely reflects diagnostic limitations rather than true absence. Therefore, it is reasonable to expect that many cases recorded as ‘Unknown’ include underlying illness; however, the actual proportion cannot be determined from the available data. In the Australian context, wildlife hospital studies demonstrate that approximately 10% of admissions are formally recorded as disease-related [21,22,23]. However, even in these situations, diagnosis is limited by feasibility and cost–benefit. The range of diseases that affect native animals is very wide and many may present with similar clinical signs [39,40,41]. Without pre- and post-mortem analysis, including further diagnostic testing and histological examination, the proportion affected by disease is likely underestimated.

This has two key implications. First, improving data collection at the time of rescue, including consistent recording of age class (e.g., dependent young, juvenile, subadult, adult), body condition, behavior, and location, and whether an attack or injury was witnessed, would strengthen interpretation. Second, targeted investment in diagnostic testing for a subset of ‘Unknown’ cases, including necropsy where appropriate, could help identify underlying causes. A pilot program could prioritize threatened species with high numbers of ‘Unknown’ records, such as gliders and microbats, and selected companion-animal attack cases. This would help determine whether animals had underlying disease, trauma, poor body condition, toxin exposure, or other pre-existing vulnerabilities that may have contributed to rescue or increased susceptibility to attack. Even limited, strategic sampling each year could substantially improve the understanding of the underlying drivers.

### 4.2. Entanglement

Entanglement was the second most common reason for the rescue of threatened species. This category includes injuries caused by a range of human-made materials, including netting, wire, marine debris, and other items. As a result, it captures a broad set of everyday hazards in urban and periurban environments. Even so, the scale of the problem is clear. A substantial number of threatened animals is being injured by common materials in these landscapes.

The dominance of flying-foxes in this category likely reflects their regular movement through backyards and orchards, with fragmented canopy increasing the need to move through these areas and bringing them into contact with hazards such as fences, clotheslines, rope, wire, and fruit-tree netting. Species that fly or climb are especially vulnerable to loose loops, tensioned lines, and poorly designed netting. In these materials, animals can become wrapped, caught by wings or limbs, or trapped by the neck.

Research from Australia and overseas shows that entanglement is driven mainly by the presence, design, and placement of materials—not by animal ‘error’. The same types of human-made materials repeatedly cause injury across species and regions [42,43,44]. This means the hazard is predictable and preventable. Within this category, entanglement—other, netting, and marine debris all showed significant increases over the study period, while wire did not. This suggests that risk from multiple human-made materials is increasing. This is concerning because many of these injuries are technically easy to prevent.

Prevention should be tailored to the type of entanglement material and setting. In urban and periurban areas, particularly backyards, orchards and gardens, prevention includes using wildlife-safe netting with small mesh that does not trap heads or limbs, keeping netting tightly secured, removing loose loops such as rope, string or clothesline hazards, and replacing barbed or loose wire fencing with wildlife-friendly designs [44]. For marine debris, prevention requires different actions, including reducing plastic and fishing-line waste, improving retrieval and disposal of waste, removing discarded netting or rope from coastal and estuarine environments, and supporting community clean-up and hazard-reduction programs [42,45]. Although these subcategories differ in setting and prevention pathway, they share a common mechanism: wildlife becoming trapped, wrapped, or injured by human-made materials.

Public awareness is also critical. Many entanglements occur because people are unaware of how easily loose or poorly installed materials can trap animals. Clear guidance—particularly before fruiting seasons (when backyard fruit trees are producing fruit) or periods of high wildlife activity—can reduce risk by encouraging safer netting practices, removal of hazardous materials, and regular property checks. Preventing entanglement therefore depends on both safer materials and informed human behavior.

### 4.3. Weather Event

Weather events associated with extreme heat were the third-most common reason for the rescue of threatened species. However, it affected only a small number of species and largely involved the Gray-headed Flying-fox. Survival was extremely low, making climatic stress one of the most deadly causes of rescue. Extreme heat is known to cause illness and mass deaths in flying-foxes, and they begin to suffer from severe heat stress when ambient temperatures reach 38 °C to 40 °C. When temperatures surpass 42 °C, their natural cooling behaviors fail, leading to rapid and fatal hyperthermia [46,47,48]. These events lead to dehydration, overheating, organ failure, and collapse [46,47,49]. The very low release rate (1.4%) reflects the irreversible multi-organ failure associated with extreme heat in mammals [50,51,52]. Drought contributes indirectly to mass deaths by lowering resilience to survive a sudden heatwave.

Rescues in this category did not occur evenly over time. Most years had few or no cases. Instead, numbers spiked sharply during major heat events, particularly in 2016–2017 and 2019–2020. This shows that climatic stress acts in sudden, intense episodes rather than as a constant background threat. These heat events also coincided with rises in other categories, including orphaned young and animals recorded as ‘Unknown’. This suggests that extreme heat does more than cause direct adult deaths. It also contributes to orphaning, displacement, and severe health decline in animals whose exact cause of collapse cannot be clearly identified. In this way, heat stress and drought have widespread impacts on wildlife populations.

Although most cases involved flying-foxes, heat likely increases risk for many other species. High temperatures reduce food and water availability and weaken animals physically [53,54]. For example, hotter and drier conditions may exacerbate risks for koalas in already fragmented landscapes, where ground movement between trees can increase exposure to cars and dogs [55,56]. Climatic stress therefore increases the risk of rescue, both directly through physiological stress and indirectly through increased exposure to other causes such as vehicle collisions and dog attacks.

Importantly, heat-related flying-fox mortality is one of the few wildlife threats that can be predicted. Flying-fox camps (large daytime roosting sites where hundreds to thousands of bats gather in trees) are fixed in location, and extreme heat events can be forecast in advance. This creates a clear opportunity for prevention. Early warning systems, trained responders, and camp-specific heat response plans can be activated before mortality escalates [46,47,48]. Habitat quality also matters. Camps with dense canopy cover, layered vegetation, and access to water can reduce heat exposure and improve survival [57]. Strengthening these features can help camps cope better with extreme heat over time. During extreme heat events, cooling measures such as sprinklers, misting, and added shade have been used [47,58]. However, their effectiveness varies [47]. Poorly designed misting can increase humidity and worsen heat stress, and sprinkler systems may only be effective under certain conditions [47].

Even modest reductions in heat-related mortality could prevent large numbers of rescues during major events. The sharp but predictable spikes seen in some years suggest that targeted investment in early warning systems, trained responders, and improving camp habitat conditions offers one of the clearest opportunities to reduce large-scale wildlife deaths.

### 4.4. Orphaning and Dependent Young

Orphaning was the fourth most common reason for the rescue of threatened species. High numbers of dependent young rescues are common in wildlife rehabilitation systems worldwide. Across birds and mammals, juveniles often account for a substantial proportion of admissions, frequently representing 20–40% of annual rescues [21,22,34,35]. Seasonal peaks in orphan admissions have been reported across Australian wildlife rehabilitation studies, particularly during spring breeding periods [17,21,22,23,25].

Importantly, the label ‘orphan’ does not always indicate confirmed parental death. In many cases, it reflects uncertainty at the time the animal was discovered. Young animals may be collected because they are found alone, on the ground, outside a nest or pouch, or temporarily unattended, even when parental care has not ceased [34,59]. As a result, this category can include animals that have genuinely lost their parents, those rejected by parents, and those collected because observers were unsure whether they had been abandoned.

Because uncertainty plays a role in some rescues, prevention opportunities arise when members of the public first encounter young animals. Some rescues occur when juveniles are discovered during peak breeding periods and appear vulnerable while moving more independently. Studies of Australian wildlife clinics have documented unnecessary rescue as a recurring cause of admission, particularly for birds [23,25]. Clear public guidance—including wildlife hotlines, simple decision guides, and targeted seasonal messaging—has therefore been recommended as a practical way to reduce avoidable admissions while still protecting genuinely at-risk young [23,60]. Improving early advice may reduce unnecessary captivity and allow limited rehabilitation resources to focus on animals in genuine need.

In some species, early-life mortality is shaped by normal reproductive biology rather than external threats alone. Many dasyurid marsupials, such as the brush-tailed phascogale (*Phascogale tapoatafa*), produce more newborns than they can physically raise [61]. Survival is immediately limited by the number of teats available for attachment, and surplus young die very early. Several bird species show similar patterns. Masked Owls and Powerful Owls typically lay two or more eggs but often raise only one chick to fledging [62,63]. Eggs hatch at different times, and when prey is scarce, parents may selectively feed the stronger chick, allowing the younger, weaker chick to die [62]. These examples show that juvenile mortality in threatened species may result from normal biological processes or environmental conditions, highlighting the importance of understanding what is typical for each species when interpreting rehabilitation data.

Interpretation of orphaning records in the NSW Wildlife Rehabilitation Data Dashboard is further limited by the absence of age information. The dataset does not distinguish dependent young, juveniles, subadults, and adults. Without age data, it is not possible to determine whether admissions primarily involve dependent young or juveniles, which are often more vulnerable to injury, separation from parents, and predation. In species where juvenile mortality is naturally high or strongly influenced by environmental conditions, some rescues recorded in rehabilitation data may reflect background mortality rather than additional population-level impacts. Because age structure cannot be assessed, the broader population consequences of orphan admissions cannot be determined from these data alone. It is recommended that future data collection include species-appropriate age classification, such as dependent young, juvenile, subadult, and adult where relevant.

In the present dataset, more than 90% of orphan rescues involved flying-foxes. In this species, dependent young may enter care following adult mortality during extreme heat events, which can leave pups orphaned in roost sites [48,54]. Consistent with this, orphan admissions increased during periods of extreme heat in the present dataset. Orphaning in flying-foxes therefore often occurs as a secondary consequence of earlier environmental stress. Reducing adult mortality—particularly during extreme heat events—would likely reduce both adult losses and subsequent orphan admissions. Disturbance at camps—such as tree trimming, noise, smoke, or human harassment—and direct threats to breeding females, such as entanglement in netting or wire, electrocution, or vehicle strike, may also contribute to orphaning in some cases.

### 4.5. Unsuitable Environment

Unsuitable environment was the fifth most common reason for the rescue of threatened species. Cases recorded in this category involve animals found in places where they cannot safely survive—for example, on roads, in buildings, in backyards, or in highly modified areas that lack food, shelter, or safe movement routes. These situations are predictable outcomes of urban growth, vegetation clearing, and fragmented habitat. As cities expand, tree cover and habitat corridors are removed, forcing wildlife into human-dominated spaces where the risk of injury, displacement, or human intervention increases.

Research shows that human disturbance and urbanization change how wildlife behave [64,65]. Many species shift activity to quieter times, move through smaller habitat areas, and may be forced to travel on the ground when tree cover is reduced or habitats become fragmented [66]. These changes increase energy use and exposure to hazards such as roads, dogs, cats, fences and entanglement. Urban expansion is also replacing remnant habitats with development [67,68]. In Australia, vegetation loss and periurban growth are reducing canopy cover and landscape connectivity [69,70]. As a result, threatened species are more often pushed into roads, backyards, and other high-risk areas, where the chance of injury or death is higher.

Prevention for this cause of rescue lies largely in landscape design rather than rescue response. Retaining tree canopy, protecting connected habitat corridors, restoring vegetation along waterways, and designing wildlife-sensitive roads and urban infrastructure can reduce forced ground movement and lower exposure to hazards [71]. However, small improvements can be overwhelmed if broader planning systems continue to allow clearing and fragmentation [67,72].

Human responses also shape these rescues. Animals are sometimes reported or removed because people fear they may be dangerous, sick, or aggressive, or because they are perceived as a nuisance in yards or buildings. Research shows that public attitudes, trust, and access to advice influence whether wildlife encounters escalate into harm to wildlife [14,73]. Clear guidance and support for coexistence can reduce unnecessary removal and improve outcomes. Prevention therefore depends on designing and managing landscapes, so animals are less often pushed into harm in the first place.

### 4.6. Motor-Vehicle Collision

Motor-vehicle collision was the sixth-most common reason for the rescue of threatened species. Outcomes are poor, with fewer than one in five animals struck by vehicles ultimately released. This matches findings from wildlife hospitals across Australia, where vehicle strike is repeatedly identified as a leading cause of severe injury and death [21]. Importantly, rescue data capture only animals that survive long enough to be found and transported to care. Many animals die at the scene and are never recorded. Rescues therefore represent only the visible part of a much larger number of deaths [74,75].

Research in road ecology shows that wildlife–vehicle collisions are not random. Risk increases where roads cut through the habitat, especially near waterways, narrow strips of vegetation that animals use to move between habitats, or movement routes between feeding and shelter [15]. Risk also rises at predictable times—such as dusk and night, during breeding or when young animals leave their home area to find new territory, and during heat or drought when animals move more or are forced to travel at ground level [76]. Koalas clearly illustrate this pattern. In fragmented landscapes, they must descend to the ground to move between trees, which greatly increases the risk of vehicle strike, especially during breeding, when young animals move away to establish their own range, and where roads separate patches of habitat [55].

The predictability of road trauma makes it one of the most preventable threats in the dataset. Decades of research show that prevention strategies are most effective when targeted to known hotspot locations rather than applied broadly [77]. Physical prevention measures are consistently identified as the most effective tools. A global review found that combining wildlife fencing with crossing structures reduced the roadkill of large mammals by 83% [78]. Best-practice guidance emphasizes that fencing and crossing structures must be implemented together, as fencing alone can block animal movement if not paired with safe crossing opportunities [78]. These measures work because they physically prevent animals from accessing the road while allowing animals to move safely between habitats.

Where large-scale infrastructure (such as wildlife fencing and dedicated crossing structures) is not possible, speed management and driver behavior become critical. Even small reductions in vehicle speed can substantially reduce how serious crashes are and overall accident rates [79,80]. Electronic or sensor-activated warning signs linked to wildlife hotspots have been associated with marked behavioral change, with one controlled study reporting a reduction in collision likelihood from 36% to 2% when enhanced signage was present [81]. However, reduced speed limits alone have shown mixed effectiveness in some contexts [82]. Overall, evidence suggests that speed management is most effective when combined with targeted warning signs, enforcement, and temporary lower speed limits during high-risk periods such as dusk or breeding season, rather than relying on permanent roadside signs alone [77,83].

Recorded rescues for motor-vehicle collision increased over the study period. This may reflect increasing road-related pressures, changes in reporting, or both. This trend is consistent with expanding road networks, increasing traffic volumes, periurban growth, and continued loss of connected habitat that forces animals to cross roads more often [15,75]. From a prevention perspective, road trauma stands out. It affects many species, leads to severe outcomes, occurs in predictable locations and times, and can be substantially reduced using targeted solutions that research shows work. Strategic installation of fencing and crossing structures at high-risk sites, combined with dynamic speed management and enforcement during peak movement periods, offers measurable reductions in mortality. Even modest reductions at identified hotspots would prevent a substantial number of rescues, and likely avert a far larger number of unseen deaths that never reach care.

### 4.7. Companion-Animal Attacks in Context

Public debate in Australia often highlights pet cats as major contributors to wildlife decline. In this threatened species rehabilitation dataset, however, companion- animal attacks, including both cats and dogs, made up only a small proportion of rescues. All rescue categories are shaped by immediate survival, detection, accessibility, and human reporting behavior. These factors may affect not only the proportion of rescues associated with predation by dogs and cats but all causes of rescue, and their relative direction and magnitude cannot be estimated from the available data. The data should not be interpreted as estimates of total wildlife injury, illness, mortality, predation pressure, or ecological impact in the field. Predation studies and rescue datasets measure different processes: predation studies estimate prey removal or hunting activity, whereas rehabilitation records measure only animals found alive and brought into care. It is not possible to determine from these rescue records whether any recorded rescue category is over- or under-represented relative to actual morbidity or mortality in the field. The results show only that companion-animal attacks formed a small proportion of recorded threatened species rescues in highly disturbed environments.

Dog-related rescues were more than three times as frequent as cat-related rescues, with cat attacks accounting for only 0.6% of all threatened species rescues, and 1.2% of recorded threatened bird rescues. Cat attacks were also proportionally less common in threatened species rescue records (0.6%) than in all wildlife rescue records statewide (1.8%). This may reflect that most free-roaming cats in highly disturbed environments are in low socioeconomic residential areas; these urban areas may have a smaller proportion of threatened species relative to common wildlife species [84,85]. Although cat attacks represented a small proportion of recorded rescues, their occurrence across 52 threatened species means that localized or species-specific impacts may still warrant targeted investigation, particularly where records cluster among vulnerable species or small populations. Combined dog and cat attacks made up 2.6% of threatened species rescues, while bird attacks accounted for a further 0.8% of all threatened species rescues. Similar patterns are reported in other Australian wildlife hospital and emergency-response datasets, where vehicle strike, orphaning, and environmental displacement, and not cat attacks, were common reasons for hospital admission or rescue [18,21,22,24,25].

Dog- and cat-related rescues affected species in predictable ways. Dog-related rescues most often involved larger mammals such as koalas and Gray-headed Flying-foxes, likely when illness, dehydration, heat stress, or habitat fragmentation forced them onto the ground. Similarly, cat-related rescues more often involved smaller tree-dwelling and flying species, including gliders, microbats, and small possums which may have been found at ground level because of illness, injury, displacement or fragmented habitat. For most species represented in this dataset, however, companion-animal attacks were not the leading recorded reason for rescue (Table A1, Table A2 and Table A3). For example, in the Little Bent-wing Bat, unsuitable environment (37%) and unknown cause (30%) were the leading reasons for rescue, with cat attack ranking third (15%) (Table A1). Species such as gliders and small possums are primarily arboreal and do not usually spend time on the ground [86]. When they are found on the ground, it is often because they are moving between disconnected trees in fragmented landscapes, or because they are injured, sick, dehydrated, or are displaced juveniles [15,87]. These situations may increase vulnerability, meaning attack records may reflect underlying illness, injury, displacement, or habitat fragmentation as well as predation risk.

Cat-related rescues may sometimes involve animals that are already vulnerable for other reasons. Birds killed by cats have been shown to have smaller spleens—a sign of reduced immune function—than birds dying from other causes [88]. Urban studies have similarly reported that birds killed by pet cats tended to be in poorer physical condition than birds killed in collisions [10]. These findings should be interpreted cautiously, but they are consistent with the broader pattern that wildlife vulnerability often reflects multiple overlapping causes of harm which increase susceptibility to cat predation in highly disturbed environments.

Feral cats have been shown to have substantial impacts on Australian wildlife, based on analyses of diet, population estimates, and the modeling of predation rates [89,90]. These findings have often been used to infer the impact of pet cats [7,8,91], even though pet cats live and behave very differently from feral cats. Feral cats survive by hunting in natural landscapes, whereas pet cats are owned, fed by people, and mostly live in urban and periurban areas.

Importantly, the wildlife in this dataset were found and rescued by people, usually in or near human settlements or places people regularly visit. The dataset is therefore most informative about recorded wildlife injury and illness in human-frequented and highly modified environments. It should not be extrapolated to infer the impacts of feral cats in natural environments or remote, less disturbed landscapes where detection, rescue probability, prey communities, and cat behavior differ substantially. In this dataset, the cats involved in recorded attacks are most likely pet cats, semi-owned cats (fed by people who do not perceive they are the owner), or stray cats living close to houses or businesses, rather than feral cats living in remote bushland. These differences matter when interpreting risk. In this dataset, attacks by companion animals made up a much smaller share of recorded rescues than human-associated factors such as climatic stress, displacement, entanglement, and road trauma.

Several urban ecology studies indicate that the effects of pet cats are context-dependent and should not be assumed to operate uniformly across all urban settings. In established suburban environments, prey composition studies have found that house cats often act as opportunistic predators: introduced mammals, particularly mice, rats and rabbits, constituted 97–98% of recorded mammal prey, while most reptile prey were small lizards, skinks or Asian house geckos. Fewer cats caught birds, and nearly half of recorded bird prey were introduced species, while amphibians were rarely recorded [84,92,93,94]. Other urban studies have found no significant relationship between cat density or cat restrictions and the diversity or abundance of selected bird or mammal communities [95,96]. Instead, housing density, distance to bushland, size of nearby bushland, vegetation density, habitat quality or landscape context were stronger predictors of wildlife occurrence [95,96,97,98]. These findings do not show that cats do not affect wildlife. Rather, they support a more nuanced interpretation: in highly modified urban environments, habitat quality, vegetation structure, landscape configuration and the local abundance of native and introduced wildlife populations more strongly influence measurable population-level effects than does cat predation.

Where companion-animal attacks on threatened and endangered species cluster in particular suburbs or periurban areas—areas on the edge of cities where housing meets bushland—targeted local responses are likely to be most effective. Prevention pathways differ for dogs and cats. For dog-related risk, prevention should focus on areas where susceptible mammals, particularly koalas, move through backyards or periurban habitat. Dog attacks on koalas are commonly reported in backyards and at night, and guidance on prevention should include keeping dogs indoors or securely confined at night and on a lead in koala habitat areas [99]. In these areas, separating dogs from known wildlife movement routes and providing safe escape routes from backyards may also reduce attacks.

For cat-related risk, practical localized measures may include supporting cat owners with containment such as cat-proof fencing, as well as behavioral approaches such as night-time feeding to encourage cats indoors at night. While mandated containment is often proposed, 89% of pet cats in NSW are already contained at least at night (when many threatened mammals are active), and many free-roaming cats are unowned or semi-owned and therefore do not have an identified owner who can implement containment [12]. Targeted responses are likely to be more useful than broad, non-specific approaches where recorded cat-related rescues or high densities of free-roaming cats are identified in particular local areas. An effective management option is sustained, high-intensity sterilization-based management of free-roaming cats, particularly where programs are linked to caregivers, including regular feeding, monitoring, adoption or transition-to-ownership pathways, and support for cat owners. Several long-term programs have reported substantial reductions in cat numbers over time, including declines of 54–99% across multiple sites and timeframes [85,100,101,102,103,104,105]. However, these outcomes depend on sufficient intensity, duration, resourcing, monitoring, and early sterilization of immigrant cats. Importantly, low-intensity programs in open populations usually fail to reduce cat numbers [106]. Recent evidence based on isotope analysis of hair samples indicates that sterilization combined with consistent provision of cat food results in a shift from consuming wild prey to a diet resembling those of indoor cats, relying mostly on commercial food [107]. These programs are most relevant where free-roaming cat densities are high, where threatened species are present, or where financial and housing constraints limit containment.

Disease risk is sometimes raised as a concern in relation to free-roaming cats. These risks are pathogen-specific and are influenced by age, diet, breeding status, veterinary care, and management [108,109,110]. For example, *Toxoplasma gondii* oocyst shedding occurs mainly in young cats during primary infection, with re-shedding by adult cats considered uncommon [111]. Unmanaged breeding populations can therefore continually produce susceptible kittens, while sterilization reduces the proportion of young immunologically naïve cats which assists in reducing the risk of environmental contamination [112]. Adequate feeding also reduces the need to consume prey to meet nutritional requirements and therefore, reduces the risk of consuming prey infected with tissue cysts, which in susceptible cats, results in oocyst shedding 3 to 10 days later. Vertebrate remains were found more frequently in the scats of poorly fed cats than adequately fed cats [113] and provision of nutritionally complete food reduced prey killed by pet cats [114]. Because a combination of sterilization and feeding cat food resulted in a change from consumption of prey to predominantly cat food, it would be expected to also reduce environmental contamination with toxoplasmosis oocysts [107]. Veterinary care, parasite control, and preventing access to raw meat, offal, and carcasses can further reduce transmission risks, including risks associated with sarcocystosis from dogs and cats [115]. These considerations support the structured management of free-roaming cats in highly disturbed environments, rather than unmanaged breeding populations.

Public discussion often concentrates on the impacts of pet cats, yet wildlife mortality in NSW is influenced by a wide range of human decisions and management activities beyond companion animals. For example, evidence presented to the 2025 NSW parliamentary inquiry into licenses to harm native animals indicated that more than 485,000 native animals (including kangaroos, wombats, possums, galahs, and sulphur-crested cockatoos) were authorized to be harmed or killed under license during 2025 alone [116]. This dwarfs the approximately 90,000 wildlife rescues occurring annually in NSW. While these licenses serve specific management purposes, their scale shows that wildlife mortality in NSW is shaped by a wide range of human decisions and activities beyond companion animals. Placing cat-related rescues in highly modified environments in this broader context is important for balanced and evidence-based conservation policy.

### 4.8. Human Welfare Implications: Compassion Fatigue and Burnout in Wildlife Rescue

The effects of the harm resulting in wildlife rescue are not limited to animals. They also affect the people who care for them. When large numbers of threatened animals enter care through severe and often preventable events, wildlife rescuers and rehabilitators are repeatedly exposed to suffering, death, and loss [117,118,119]. This burden becomes especially intense during mass rescue events, such as extreme heat affecting flying-foxes. In this NSW dataset, rescues due to weather events associated with extreme heat, drought and fire were concentrated in short event periods rather than being evenly distributed across years. These sharp peaks suggest that acute workload and emotional burden for rescuers are likely to be greatest during episodic mass rescue events, especially when associated with very poor survival, rather than being driven only by steady background rescue activity.

Research across animal-care professions shows that heavy workloads, limited resources, and frequent poor outcomes increase the risk of compassion fatigue, secondary traumatic stress, and burnout [118,120,121]. Studies of Australian wildlife carers report similar patterns. Emotional exhaustion and grief rise when carers manage large numbers of animals, provide long-term care, and face repeated deaths [117,118,119]. These pressures are often greater in wildlife rescue because the sector relies heavily on volunteers and has limited access to formal mental health support [118].

Reducing preventable rescues can therefore benefit both animals and people. Preparing for extreme heat events at flying-fox camps, reducing road and entanglement hazards, and limiting habitat loss can lower the number of animals arriving in critical condition. Fewer mass rescue events and fewer severe cases mean less repeated exposure to trauma for carers.

This human dimension is important. High levels of stress and burnout can lead to volunteers leaving the sector, reducing the long-term capacity of wildlife rescue systems. Preventing predictable harm therefore has a double benefit: it reduces suffering for wildlife and helps protect threatened species by reducing the number of animals entering care. Protecting wildlife also means protecting the workforce that supports them.

### 4.9. Limitations and Value of the NSW Dashboard

This study has several important limitations. The NSW Wildlife Rehabilitation Data Dashboard includes only animals that were found, rescued, and reported, so it does not represent all wildlife injury and mortality across the state. Whether an animal enters care depends on immediate survival, detection, accessibility, proximity to people, and the motivation and capacity of the finder to seek assistance. These factors are likely to vary across all recorded causes of rescue. For example, animals struck by vehicles may be more visible but may also die before rescue; animals affected by habitat loss or displacement may die unseen; and predation, disease or toxin exposure may go undetected unless the animal is found alive. The dashboard data do not allow the direction or magnitude of these biases to be estimated. Therefore, the results should be interpreted as patterns in recorded rescue burden, not as cause-specific estimates of total mortality or ecological impact.

The dataset is also shaped by differences in how likely different species are to be found and rescued. Animals that are more visible, easier to handle, or found in areas with more people or rescue services are more likely to appear in wildlife rescue records. This limitation is particularly relevant to small-bodied prey species, including small reptiles, amphibians, birds, and mammals, which may be killed or remain undetected before they can enter the rescue system. Similarly, records are more likely to arise from places where people live, work, travel or recreate. The findings are therefore most relevant to wildlife morbidity and mortality in human-frequented and highly modified environments, and should not be extrapolated to natural environments or remote, less disturbed landscapes. The mammal results should also be interpreted with recognition that Gray-headed Flying-foxes and Koalas contributed a large proportion of mammal records; therefore, patterns for mammal rescues as a group should not be assumed to apply equally to all threatened mammal species.

Recorded reasons for rescue also reflect what could be identified at or soon after rescue. The dashboard does not include detailed case-level information such as veterinary findings, body condition, diagnostic testing, post-mortem results or confirmation of whether an attack was witnessed. These limitations affect interpretation across all categories, including vehicle collision, entanglement, environmental displacement, disease and companion-animal attacks. For all categories, including companion-animal attacks, the recorded proportions should be interpreted only as recorded rescue events, not as estimates of total morbidity, mortality, predation pressure, or ecological impact.

The aggregated structure of the dashboard also limited the statistical analyses that could be undertaken. Although suburb/locality-level location information is available, the dataset does not provide precise coordinates, standardized survey sites, reporting effort, observer effort, search effort, detection histories, population denominators, habitat covariates, or repeated site-level observations. Therefore, formal spatial modeling, occupancy or detection modeling, hierarchical modeling, and correction for reporting effort were not appropriate. Linear regression analyses of annual counts were used only to identify exploratory patterns in recorded rescue numbers over time, and should not be interpreted as estimates of true incidence, population-level risk, or ecological drivers. Future analyses would be strengthened if case-level records, standardized spatial information, effort measures, and other environmental factors were made available.

Temporal trends should also be interpreted cautiously because annual rescue numbers may be influenced by changes in reporting effort, public awareness, volunteer capacity, rescue-service access, urbanization, and human–wildlife overlap. The dashboard does not provide the information needed to separate these factors from true changes in wildlife morbidity or mortality. Therefore, increases over time are interpreted as increases in recorded rescue burden, not as direct evidence of increased ecological threat. Temporal trends also require cause- and species-specific interpretation. Some increasing categories were largely driven by a single species. The disease chlamydia was almost entirely associated with Koalas, while electrocution was overwhelmingly associated with Gray-headed Flying-foxes. These patterns should therefore not be interpreted as broad cross-species increases in disease or electrocution. Instead, they are useful as they point to targeted prevention and management opportunities for particular species and contexts, including koala disease management and flying-fox interactions with urban and periurban electrical infrastructure.

Spatial analyses were also beyond the scope of the present study. Although the dashboard provides broad area-level location information, it does not provide the spatial resolution or associated environmental covariates required for hotspot mapping, urban–rural comparison, or habitat association analyses. Future research using case-level spatial data, standardized location fields, land-use layers, habitat covariates, and reporting effort measures could build on this statewide summary to identify spatial hotspots and landscape-level predictors of threatened species rescues.

Despite these limitations, the NSW Wildlife Rehabilitation Data Dashboard is a valuable statewide resource. It brings together large numbers of records over many years and makes it possible to identify broad patterns in wildlife rescue, causes of admission and outcomes. These data are particularly useful for identifying common and preventable causes of recorded rescue for vulnerable species, guiding targeted prevention efforts, and highlighting where improved data collection, veterinary assessment, or diagnostic sampling would strengthen future interpretation and prevention. Similar wildlife rehabilitation dashboards in other Australian states would strengthen the evidence-base for conservation and support more effective, targeted policies to protect threatened species and other wildlife.

## 5. Conclusions

This study provides the first statewide summary of threatened species rescues in New South Wales. The findings show that threatened animals entering care were concentrated within a small number of recorded rescue categories. The six most common were Unknown, Entanglement, Weather Events, Abandoned/Orphaned, Unsuitable Environment, and Motor-Vehicle Collision. Together, these causes accounted for 68% of all recorded threatened species rescues. The large Unknown category should be interpreted cautiously because it indicates that the underlying cause could not be confirmed at the time of rescue, often because animals were already in severe condition, and likely included under-detected disease, trauma, environmental stress, toxin exposure, or multiple overlapping conditions. Funding for in-depth diagnostic testing is recommended for a subset of wildlife where the cause of rescue is Unknown, particularly for highly vulnerable species and where Unknown is over-represented as a recorded cause of rescue for threatened species.

Among cases where a cause could be identified, many recorded rescues were associated with pressures linked to human-modified landscapes, environmental stress, habitat loss and fragmentation, hazardous materials, roads, and urban infrastructure. Companion-animal attacks accounted for a small proportion of recorded rescues overall, with dog-related rescues being more than three times as common as cat-related rescues. These findings should not be interpreted as estimates of total wildlife mortality, predation pressure, or ecological impact. Rather, they identify the recorded causes most commonly represented among threatened animals entering care.

Rescue records can help identify practical prevention opportunities within the rehabilitation system. The findings support targeted action to prepare for extreme heat events, protect and reconnect habitat, reduce entanglement hazards, improve road safety in wildlife hotspots, and provide clear public guidance when young animals are found. Landscape-level design, including retaining canopy cover, reconnecting habitat corridors, and integrating wildlife-safe road planning, is likely to be important for reducing recorded rescues linked to unsuitable environments and motor-vehicle collisions.

Expanding rescue capacity will not address the underlying conditions that lead animals into care. Prevention-focused strategies can reduce recorded rescue burden, improve outcomes for threatened species, and reduce pressure on wildlife rescuers and rehabilitators. These strategies are most useful when applied as part of integrated urban wildlife management rather than as isolated single-threat responses. Recognizing and addressing the most common and preventable recorded causes identified in this study can therefore support more targeted conservation planning. However, rehabilitation records do not measure the full scale of wildlife injury, mortality, or ecological impact, even in highly disturbed environments. They should also not be extrapolated to the morbidity or mortality of threatened species in remote or natural environments, where feral cats are documented to have significant negative impacts. Overall, these findings support an integrated approach that combines extreme heat preparedness, habitat retention and reconnection, wildlife-safe road planning, reduction in entanglement hazards, improved public guidance, targeted diagnostic investigation, and locally targeted companion-animal management where rescue records indicate specific risk.

## Figures and Tables

**Table 1:** Standardized ‘Reason for Rescue’ categories used in this study (from the NSW Wildlife Rehabilitation Data Dashboard).

Major Rescue Reason Group	Individual Categories
Unsuitable environment	Unsuitable environment; Stranded/haul-out ^1^
Dependent young	Abandoned/orphaned; Fallen from nest/tree; Parent taken into care
Entanglement	Other; Wire; Marine debris; Netting
Weather event	Drought; Storm; Fire; Extreme heat; Flood; Other
Disease	Other; Chlamydia; External parasite; Mange; Internal parasite
Attack	Dog; Cat; Bird; Same species; Fox; Suspected/other
Collision	Motor vehicle; Building; Other
Human-related impact	Habitat alteration/tree-felling; Human interference; Intentional harm
Other human-related causes	Fouled by substance; Ingestion of foreign object; Negative interaction; Poisoned; Electrocution; Entrapment
Unknown	Unknown

^1^ Haul-out refers to marine mammals (e.g., seals) coming out of the water onto land. In this dataset, it indicates cases where the animal’s location was considered unsafe or inappropriate and intervention was required.

**Table 2:** Threatened wildlife rescues in New South Wales (2013–2024) by major animal group, showing the number of individuals rescued, percentage of total threatened rescues, and number of threatened species represented.

Animal Group	No. of Individuals	No. of Species
Mammals	45,686 (87.1)	35
Birds	4436 (8.5)	91
Reptiles	2337 (4.4)	28
Amphibians	16 (<0.1)	4
**Total**	**52,475**	**158**

**Table 3:** The 10 most common reasons for rescue across all wildlife species (*n* = 967,492) in New South Wales (2013–2024), showing number of individuals and percentage of total rescues.

Rescue Reason	No. of Individuals (%)
Unknown	475,165 (49.1)
Motor-vehicle collision	122,886 (12.7)
Unsuitable environment	65,575 (6.7)
Abandoned/orphaned	44,340 (4.6)
Fallen from nest/tree	38,211 (3.9)
Entanglement ^1^	28,174 (2.9)
Collision—other	23,999 (2.5)
Parent taken into care	23,786 (2.4)
Dog attack	20,130 (2.1)
Cat attack	17,272 (1.8)
**Total**	**793,963 (82.1)**

^1^ Breakdown of entanglement subcategories as follows: other, *n* = 14,524; wire, *n* = 9012; marine, *n* = 3199; netting, *n* = 1439.

**Table 4:** The 10 most common reasons for rescue among threatened species in New South Wales (2013–2024), showing number of individuals rescued, percentage of all threatened species rescues (*n* = 52,475), number of threatened species affected, and proportion released alive.

Rescue Reason	No. of Individuals (%)	No. of Species Affected	% of Individuals Released
Unknown cause	11,486 (21.9)	140	23.5
Entanglement ^1^	6101 (11.6)	49	32.4
Weather event—extreme heat	5985 (11.4)	5	1.4
Abandoned/orphaned young	5326 (10.1)	50	21.3
Unsuitable environment	3820 (7.3)	86	51.5
Motor-vehicle collision	3050 (5.8)	83	19.8
Disease—chlamydia	2458 (4.7)	2	24.7
Electrocution	2121 (4)	8	7.4
Collision—other	1398 (2.7)	61	21.5
Weather event—fire	1359 (2.6)	10	13.7
**Total**	**43,104 (82.1)**	**154**	**22.6**

^1^ Breakdown of entanglement subcategories as follows: other, *n* = 3222; wire, *n* = 2340, marine, *n* = 151; netting, *n* = 388.

**Table 5:** Animal attack-related rescues among threatened species in New South Wales (2013–2024), showing the number of individuals, percentage of all threatened species rescues (52,475), number of threatened species affected, and proportion released alive.

Attack Type	No. Individuals	% of All Threatened Species Rescues	No. Threatened Species Affected	% of Individuals Released
Dog attack	1032	2.0	36	26.3
Suspected/unidentified attack	516	1.0	49	32
Bird attack	401	0.8	47	16.5
Cat attack	311	0.6	52	21.5
Same-species attack	37	0.1	5	48.6
Fox attack	5	0.01	4	20
**All animal attacks**	**2302**	**4.4**	**92**	**25.5**

**Table 6:** The four most common reasons for rescue for threatened species in selected animal groups in New South Wales (2013–2024), showing leading recorded reasons and number of individuals rescued.

	Most Common Reasons for Rescue (*n*)			
Animal Group/Focal Species	1st	2nd	3rd	4th
Threatened mammals (all species)	Unknown cause (8551)	Weather event (5983)	Entanglement (5875)	Abandoned/orphaned young (5152)
Koala (*Phascolarctos cinereus*)	Disease—chlamydia (2450)	Unknown cause (2401)	Motor-vehicle collision (1875)	Unsuitable environment (1774)
Gray-headed Flying-fox (*Pteropus poliocephalus*)	Weather event (5946)	Unknown cause (5701)	Entanglement (5569)	Abandoned/orphaned young (4831)
Threatened birds (all species)	Unknown cause (1773)	Motor-vehicle collision (619)	Unsuitable environment (537)	Collision—other (348)
Threatened Reptiles (all species)	Unknown cause (1150)	Stranded/haul-out events (596)	Disease—other (141)	Habitat alteration/tree felling (75)

**Table 7:** Trends in numbers of threatened species rescues in New South Wales (2013–2024), showing direction of change over time and associated *p*-values for major animal groups and rescue reasons. *p*-values are from linear regression analyses testing whether annual rescues changed over time.

**Taxonomic Group**	**Trend Over Time**	***p*-Value**
Mammals	No significant trend	>0.05
Birds	Increasing	<0.01
Reptiles	No significant trend	>0.05
Amphibians	No significant trend	>0.05
**Cause of Rescue**	**Temporal Trend**	***p*-Value**
Unsuitable environment	Increasing	<0.01
Entanglement (combined)	No significant trend	>0.05
Entanglement—other	Increasing	<0.05
Entanglement—netting	Increasing	<0.01
Entanglement—marine debris	Increasing	<0.01
Entanglement—wire	No significant trend	>0.05
Motor-vehicle collision	Increasing	<0.01
Weather event	No significant trend	>0.05
Abandoned/orphaned	No significant trend	>0.05
Unknown	No significant trend	>0.05
Disease—chlamydia	Increasing	<0.05
Electrocution	Increasing	<0.05
Attack—dog	No significant trend	>0.05
Attack—cat	No significant trend	>0.05

**Table 8:** Threatened species showing significant increases in rescue numbers in New South Wales (2013–2024), with *p*-values from linear regression analyses. *p*-values are from linear regression analyses testing whether annual rescue counts for each species increased over time. *p*-values less than 0.05 were considered statistically significant.

Species (*n*)	*p*-Value	Leading Causes of Rescue (*n*)		
**Birds**				
Bush Stone-curlew (604)	<0.001	Unsuitable environment (263)	Unknown (159)	Motor-vehicle collision (63)
Powerful Owl (493)	<0.05	Unknown (232)	Motor-vehicle collision (92)	Collision—other (45)
Eastern Osprey (280)	<0.05	Unknown (99)	Unsuitable environment (35)	Attack—bird (25)
Wompoo Fruit-Dove (197)	<0.01	Collision—other (60)	Collision—building (40)	Unknown (35)
White-bellied Sea Eagle (198)	<0.05	Unknown (76)	Motor-vehicle collision (22)	Collision—other (18)
Pied Oystercatcher (84)	<0.01	Unknown (30)	Entanglement (24)	Motor-vehicle collision (9)
Red-tailed Black-Cockatoo (51)	<0.05	Unknown (34)	Motor-vehicle collision (9)	8 causes each affecting 1 individual
**Mammals**				
Greater Glider (73)	<0.05	Unknown (27)	Entanglement (16)	Abandoned/orphaned (6)
Eastern Long-eared Bat (415)	<0.01	Unknown (111)	Unsuitable environment (85)	Attack—cat (54)
**Microbats**				
Southern Myotis (97)	<0.01	Unsuitable environment (28)	Unknown (26)	Attack—cat (10)
Greater Broad-nosed Bat (65)	<0.01	Unknown (20)	Unsuitable environment (15)	Habitat alteration/tree felling (6)
Yellow-bellied Sheath tail Bat (20)	<0.05	Unknown (8)	Unsuitable environment (7)	Attack—bird (2)
**Reptiles**				
Rosenberg’s Goanna (24)	<0.05	Unknown (8)	Attack—dog (7)	Unsuitable environment (4)
Stephens’ Banded Snake (27)	<0.05	Unsuitable environment (14)	Unknown (7)	Negative interaction (3)

## Data Availability

The original contributions presented in the study are included in the article, further inquiries can be directed to the corresponding author.

## Appendix A

**Table A1:** Top reasons for rescue for selected threatened mammals (NSW Wildlife Rehabilitation Data Dashboard, 2013–2024).

Species	Total (*n*)	Top Reasons Overall—Ranked (*n*, %)
Squirrel glider	653	Entanglement–other (182, 27.9%), Unknown (112, 17.2%), Attack-Cat (63, 9.6%), Unsuitable environment (56, 8.6%)
Eastern long-eared bat	415	Unknown (111, 26.7%), Unsuitable environment (85, 20.5%), Attack-Cat (54 13.0%), Habitat alteration/Tree felling (36, 8.7%)
Brush-tailed phascogale	156	Unsuitable environment (43, 27.6%), Unknown (33, 21.2%), Cat (23, 14.7%), Abandoned/Orphaned (12, 7.7%)
Little bent-wing bat	98	Unsuitable environment, (36, 36.7%) Unknown (29, 29.6%), Attack-Cat (15, 15.3%), Attack–Suspected/Other (5, 5.1%)

**Table A2:** Threatened avian species admitted following cat and dog attacks, ranked by number of attack-related rescues.

Avian Species	Attack Rescues	Total Rescues	Top 3 Causes
**Cat Attacks**			
Rose-crowned fruit-dove	8	226	Collision—other (71), Unknown (41), Collision—building (31)
Superb parrot	5	178	Unknown (94), Collision—motor vehicle (51), Collision—other (10)
Bush stone-curlew	5	604	Unsuitable environment (263), Unknown (159), Collision—motor vehicle (63)
Wompoo fruit-dove	4	196	Collision—other (60), Collision—building (40), Unknown (35)
Superb fruit-dove	3	63	Unknown (27), Collision—other (18), Collision—building (5)
Little lorikeet	3	77	Unknown (49), Collision—other (6), Habitat alteration/tree felling (6)
Latham’s snipe	3	12	Unknown (4), Attack—other (3), Attack—cat (3)
Eastern ground parrot	2	50	Unknown (38), Collision—motor vehicle (3), Unsuitable environment (2)
Red-backed button-quail	2	10	Unknown (3), 3 causes affecting 2 individuals
White-throated needletail	2	74	Unknown (20), Unsuitable environment (11), Collision—other (7) and motor vehicle (7)
Pink cockatoo	2	213	Unknown (65), Collision—motor vehicle (16), 4 causes all affecting 2 individuals
Swift parrot	1	16	Unknown (10), Collision—motor vehicle (3), 3 causes each affecting 1 individual
Regent parrot	1	8	Unknown (3), Abandoned/orphaned (3), Collision—motor vehicle (1)
Eastern curlew	1	13	Unsuitable environment (5), Unknown (4), Attack—suspected other (2)
Black-breasted button-quail	1	4	Unknown (3), Attack—cat (1)
Regent honeyeater	1	16	Unknown (6), Attack—bird (2), Fallen from nest or tree (2), Unsuitable environment (2)
Black-chinned honeyeater	1	5	Unknown (2), Unsuitable environment (1), Attack—cat (1), Attack—bird (1)
Gray-crowned babbler	1	15	Unknown (4), Attack—bird (3), Fallen from nest or tree (2)
Barred cuckoo-shrike	1	17	Unknown (9), Parent taken into care (3), 5 causes all affecting 1 individual each
Sooty tern	1	341	Unknown (158), Event—storm (90), Unsuitable environment (45)
Powerful owl	1	493	Unknown (232), Collision—motor vehicle (92), Collision—other (45)
Eastern osprey	1	280	Unknown (99), Unsuitable environment (35), Attack—bird (25)
Pale-vented bush-hen	1	22	Unknown (7), Collision—motor vehicle (6), Habitat alteration/tree felling and Abandoned/orphaned (3 each)
Pink robin	1	2	Collision—motor vehicle (1), Attack—cat (1)
Dusky woodswallow	1	27	Unknown (12), Parent taken into care (5), Abandoned/orphaned (4)
**Total**	**53 (1.2%)**	**4436**	
**Dog Attacks**			
Bush stone-curlew	14	604	Unsuitable environment (263), Unknown (159), Collision—motor-vehicle (63)
Powerful owl	6	493	Unknown (232), Collision—motor-vehicle (92), Collision—other (45)
Rose-crowned fruit-dove	5	226	Collision—other (71), Unknown (41), Collision—building (31)
Pied oystercatcher	4	84	Unknown (30), Entanglement—marine debris (17), Collision—motor vehicle (9)
Wompoo fruit-dove	2	196	Collision—other (60), Collision—building (40), Unknown (35)
Superb fruit-dove	2	63	Unknown (27), Collision—other (18), Collision—building (5)
Superb parrot	2	178	Unknown (94), Collision—motor vehicle (51), Collision—other (10)
White-bellied sea eagle	2	198	Unknown (76), Collision—motor vehicle (22), Collision—other (18)
Sooty oystercatcher	1	17	Unknown (7), Event—storm (7), Entanglement—marine debris (2)
Eastern ground parrot	1	50	Unknown (38), Collision—motor vehicle (3), Unsuitable environment (2)
Gang-gang cockatoo	1	175	Unknown (73), Collision—motor vehicle (66), Disease—other (8)
Pale-vented bush-hen	1	22	Unknown (7), Collision—motor vehicle (6), Habitat alteration/tree felling and Abandoned/orphaned (3 each)
Little tern	1	38	Unknown (12), Abandoned/orphaned (5), Unsuitable environment and Collision—other (3 each)
Black-eared miner	1	5	Unknown (3), Attack—dog and Attack—suspected/other (1 each)
White-throated needletail	1	74	Unknown (20), Attack—bird (14), Unsuitable environment (11)
**Total**	**44 (1.0%)**	**4436**	

**Table A3:** Threatened mammal species rescued following cat and dog attacks, ranked by number of attack-related rescues.

Mammal Species	Attack Rescues	Total Rescues	Top 3 Causes
**Cat Attacks**			
Squirrel glider	63	653	Unknown (112), Entanglement—other (100), Entanglement—wire (75)
Eastern long-eared bat	54	415	Unknown (111), Unsuitable environment (85), Attack—cat (54)
Gray-headed flying-fox	29	31,277	Weather Event—heat (5943), Unknown (5398), Abandoned/Orphansed (4807)
Brush-tailed phascogale	23	156	Unsuitable environment (43), Unknown (33), Attack—cat (23)
Little bent-winged bat	15	98	Unsuitable environment (36), Unknown (29), Attack—cat (15)
Eastern pygmy possum	14	312	Unknown (159), Unsuitable environment (46), Abandoned/Orphansed (21)
Large bent-winged bat	13	133	Unknown (37), Unsuitable environment (34), Collision—other (14)
Southern myotis	10	97	Unsuitable environment (28), Unknown (26), Habitat alteration/Tree felling (15)
Large-eared pied bat	4	25	Unknown (7), Unsuitable environment (6), Attack—cat (4)
Southern brown bandicoot (eastern)	4	55	Abandoned/Orphansed (13), Collision—motor vehicle (13), Unknown (10)
Koala	4	11,720	Disease—chlamydia (2457), Unknown (2401), Collision—motor vehicle (1878)
Eastern cave bat	3	20	Unknown (11), Unsuitable environment (5), Attack—cat (3)
Eastern coastal free-tailed bat	3	184	Unsuitable environment (72), Unknown (44), Habitat alteration/Tree felling (36)
Greater broad-nosed bat	2	65	Unknown (20), Unsuitable environment (15), Habitat alteration/Tree felling (6)
Southern greater glider	2	73	Unknown (27), Entanglement—wire (9), Habitat alteration/Tree felling (8)
Eastern chestnut mouse	2	8	Unknown (4), Attack—cat (2), Dependent young and Unsuitable environment (1 each)
Corben’s long-eared bat	1	16	Unsuitable environment (11), Unknown (3), Attack cat and Attack—dog (1 each)
Common blossom bat	1	14	Attack—suspected/other (4), Disease—external parasite (2), Unknown (2)
Common planigale	1	11	Unknown (5), Unsuitable environment (3), 3 causes all affecting 1 individual
Striped-faced dunnart	1	1	Attack—cat (1)
Mountain pygmy possum	1	18	Unknown (12), Event—storm (2), 4 causes all affecting 1 individual
Red-legged pademelon	1	51	Collision—motor vehicle (26), Abandoned/Orphaned (7), Parent taken into care (7)
**Total**	**251 (0.5%)**	**45,686**	
**Dog Attacks**			
Koala	637	11,720	Disease—chlamydia (2457), Unknown (2401), Collision—motor vehicle (1878)
Gray-headed flying-fox	284	31,277	Weather Event—heat (5943), Unknown (5398), Abandoned/Orphansed (4807)
Squirrel glider	19	653	Unknown (112), Entanglement—other (100), Entanglement—wire (75)
Spotted-tailed quoll	6	100	Unknown (30), Unsuitable environment (23), Collision—motor vehicle (16)
Brush-tailed phascogale	5	156	Unsuitable environment (43), Unknown (33), Attack—cat (23)
Eastern long-eared bat	5	415	Unknown (111), Unsuitable environment (85), Attack—cat (54)
Eastern pygmy possum	4	312	Unknown (159), Unsuitable environment (46), Abandoned/Orphansed (21)
Red-legged pademelon	3	51	Collision—motor vehicle (26), Abandoned/Orphaned and Parent taken into care and Unknown (7 each)
Eastern coastal free-tailed bat	3	184	Unsuitable environment (72), Unknown (44), Habitat alteration/Tree felling (36)
Little bent-winged bat	2	98	Unsuitable environment (36), Unknown (29), Attack—cat (15)
Yellow-bellied glider	1	80	Entanglement—wire (22), Unknown (21), Entanglement—wire (10)
Southern myotis	1	97	Unsuitable environment (28), Unknown (26), Habitat alteration/Tree felling (15)
Large-eared pied bat	1	25	Unknown (7), Unsuitable environment (6), Attack—cat (4)
Corben’s long-eared bat	1	16	Unsuitable environment (11), Unknown (3), Attack cat and Attack—dog (1 each)
Black-striped wallaby	1	29	Unknown (11), Collision—motor vehicle (8), Parent taken into care (7)
Large bent-winged bat	1	133	Unknown (37), Unsuitable environment (34), Collision—other (14)
Southern brown bandicoot (eastern)	1		Abandoned/Orphansed (13), Collision—motor vehicle (13), Unknown (10)
**Total**	**975 (2.1%)**	**45,686**	
